# Do primary care physicians coordinate ambulatory care for chronic disease patients in Canada?

**DOI:** 10.1186/1471-2296-15-148

**Published:** 2014-08-30

**Authors:** Alan Katz, Patricia Martens, Dan Chateau, Bodgan Bogdanovic, Ina Koseva

**Affiliations:** Departments of Family Medicine and Community Health Sciences, College of Medicine, Faculty of Health Sciences, University of Manitoba, Manitoba Centre for Health Policy, 408-727 McDermot Ave, Winnipeg, Manitoba R3E 3P5 Canada; Department of Community Health Sciences, College of Medicine, Faculty of Health Sciences, University of Manitoba, Manitoba Centre for Health Policy, 408-727 McDermot Ave, Winnipeg, Manitoba R3E 3P5 Canada; Manitoba Centre for Health Policy, 408-727 McDermot Ave, Winnipeg, Manitoba R3E 3P5 Canada

**Keywords:** Ambulatory care, Family physician, Primary care provider, Specialist, Continuity of care, Coordinated care

## Abstract

**Background:**

Adults with chronic disease are the most frequent users of the primary healthcare system. In Manitoba, patients are allowed to seek ambulatory (outpatient) care from the provider of their choosing (primary care physician or specialist), with referrals to specialists preferred but not always required. Some patients receive their routine care from specialists. We conducted this study to determine the patterns by which adults with chronic disease access ambulatory care as a prelude to exploring the impact these patterns may have on the quality of care received.

**Methods:**

Physician claims for all visits between 2007/8-2009/10 were extracted from the Data Repository at the Manitoba Centre for Health Policy. Patients included in the analysis made at least four ambulatory visits to a primary care physician or specialist within the study period, and met the definition criteria for at least one of six chronic diseases: diabetes mellitus; congestive heart failure; mood disorders; ischemic heart disease; total respiratory morbidity; and/or hypertension. Patients were “assigned” to the physician they visited most regularly. Physician visit patterns were assessed by dividing visits into nine visit types based on the type of physician patients visited (assigned primary care physician, other primary care physician, or specialist) and whether or not they received a referral.

**Results:**

347,606 patients with 7,662,411 physician visits were included in the analysis. Most visits were to the patients’ assigned primary care physician. About 50% of the visits to specialists were by referral from the assigned primary care physician. However, 26-29% of all visits to a primary care physician were not to the assigned primary care physician, and non-assigned physicians were more likely to refer patients to specialists than assigned primary care physicians.

**Conclusion:**

The findings suggest that the current primary care system in Manitoba may not adequately support coordination of ambulatory care. Ambulatory visits to a primary care provider who is not the patient’s regular provider may represent a lost opportunity for coordination and continuity of care, and may affect the quality of care patients receive. Primary care renewal initiatives in this province should address this challenge to service provision.

**Electronic supplementary material:**

The online version of this article (doi:10.1186/1471-2296-15-148) contains supplementary material, which is available to authorized users.

## Background

The Canadian healthcare system is widely perceived to have a strong emphasis on primary care, which has been shown to be the foundation for a cost–effective healthcare system promoting better population health [1]. In an effort to bring healthcare spending under control, significant investment in primary care renewal has been made, including the development of salaried models for physician care and integration of inter-professional teams in primary care [2]. The aim of these initiatives is to create a highly functioning primary care system that will result in a healthier population and less use of expensive secondary and tertiary care. Over the last decade, almost all Canadian provinces, including Manitoba, have invested in primary care renewal [3, 4].

Patients suffering from chronic disease are frequent users of the primary healthcare system [5, 6] and are the most likely population to benefit from healthcare renewal initiatives, such as improvements in coordination of care. Coordination of care refers to ‘the delivery of services by different care providers in a timely and complementary manner in order to achieve connected and cohesive patient care’ [7], and is a core element of patient-centred primary care [8]. High quality well-coordinated care is to a large extent dependent on continuity of care, an aspect of coordination described as a longitudinal and interpersonal relationship between the patient and the provider, whereby patients and physicians are cooperatively engaged in ongoing healthcare management [9]. Continuity is often explained in terms of three dimensions: 1) relational (the patient’s experience of a continuous, caring relationship); 2) information (sharing of information between different health care providers); and 3) management (the physician aims to provide seamless, integrative healthcare service) [10, 11]. This type of care is both a fundamental component of primary care and a significant contributor to good health outcomes [12–14]. Coordinated care for chronic disease involves input from a wide range of health professionals [15], and thus requires an informed healthcare team to ensure good communication among healthcare providers and patients and to facilitate optimal care [16]. A call to action from the Canadian Academy of Health Sciences recommends that the Canadian healthcare system provide all people with chronic disease with access to assigned clinicians or teams of clinicians responsible for providing their primary care and for coordinating care with acute, specialty, and community services throughout their life spans [17].

In Manitoba, ambulatory care (non-emergency, condition-specific single visit or episodic care provided on an outpatient basis in support of primary care) is offered by primary care physicians, nurse practitioners and specialist physicians, and patients are free to seek ambulatory care from any provider of their choosing. But while primary care is the recommended route of access to advanced medical care (with a referral from a primary care physician usually required for access to a specialist [18]), Manitobans may also access specialist care via alternate routes. These routes include referral to a specialist by a primary care physician who is not the patient’s regular provider, direct patient contact with a specialist without any referral, and provision of routine care from specialists.

Little is known about the patterns of routine ambulatory care Manitoban patients with chronic disease receive from their physicians. Whether accessing specialist care via the alternative routes described above or receiving care from a “non-assigned” primary care provider has consequences on the quality of care received, for example, by disrupting coordination and continuity of care, has not been established. Therefore, the objective of this study was to examine the patterns of primary physician and specialist use by adults with specific types of chronic disease in order to assess whether their care fits within the recommended patterns of coordinated care.

## Methods

### Setting

Manitoba is a central Canadian province with a population of about 1,250,500 [19]. Just over 55% of the total population resides in the capital city of Winnipeg, and the rest live in smaller communities of 47,000 or fewer residents. The physician visit patterns of residents of Manitoba’s second largest urban community (Brandon, pop 46,061) were similar to rural Manitobans. Therefore, the study compared Winnipeg to non-Winnipeg patterns when exploring the impact of geography on service use.

### Data sources and data period

The study was conducted at the Manitoba Centre for Health Policy (MCHP) at the University of Manitoba. MCHP houses the Population Health Research Data Repository (herein referred to as the Repository). The Repository contains data derived from administrative claims collected by Manitoba Health, the Government of Manitoba department that administers the universal healthcare system within the province. These data provide comprehensive information of key interest to health planners, including person–level data such as birth and mortality, contacts with physicians and hospitals, pharmaceutical dispensing, and use of nursing homes, as well as 6-digit postal code data to derive area–level data such as region of residence. All data files in the Repository are ‘de–identified’, meaning that names and other identifying fields are not available, but unique (scrambled) identifiers are used to allow linkage across files and follow–up over time. Data in the Repository have been documented and validated extensively for this type of research [20]. Data used in this study are from the Population Health Research Data Repository (HIPC# 2010/2011-35) housed at the Manitoba Centre for Health Policy, University of Manitoba and were derived from data provided by Manitoba Health and Province of Manitoba departments of Education, Family Services & Labor, Entrepreneurship, Training & Trade, the Healthy Child Manitoba Office, and the Winnipeg Regional Health Authority. Data from the fiscal years April 1^st^, 2001 to March 31^st^, 2007 were used to determine chronic disease prevalence, and data from April 1^st^, 2007 to March 31^st^, 2010 were used for the physician visit patterns analyses.

### Study cohort

**Figure 1:**
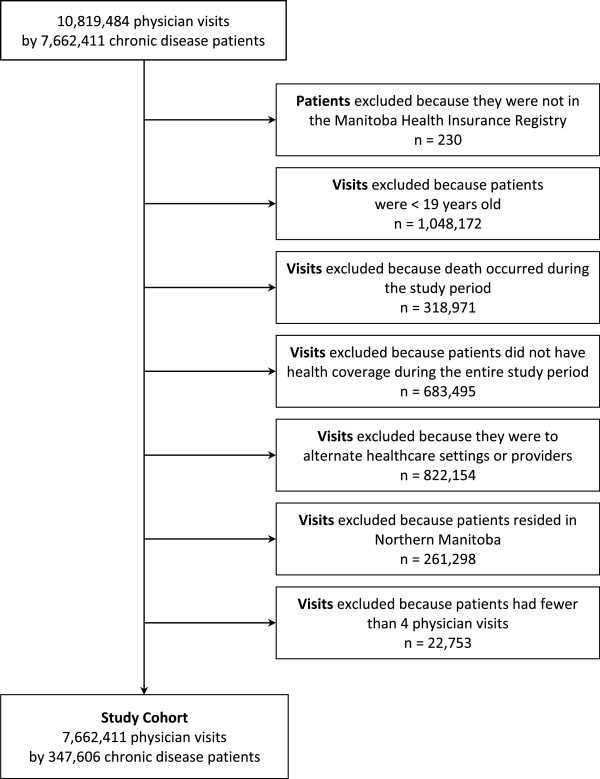
**Criteria for developing the study cohort.** Physician visit data from chronic disease patients was obtained from the Repository. Patients with one or more chronic disease(s) were included in the study if they met the following inclusion criteria: they were in the Manitoba Health Insurance Registry, were at least 19 years old, had lived in Manitoba during the entire study period, and had made at least 4 ambulatory visits to primary care physicians or specialists during the 3-year study period.

Development of the study cohort is summarized in Figure 1. We identified all physician visits during the three-year study period by individuals who met the definition criteria for at least one of six chronic diseases: diabetes mellitus; congestive heart failure; mood disorders; ischemic heart disease; total respiratory morbidity; and/or hypertension (listed in Additional file 1). Individuals were included in the study if they were in the Manitoba Health Insurance Registry, had Manitoba health coverage throughout the study period, were 19 years of age or older at the start of the study period, and had made at least four ambulatory visits to a primary care physician or specialist (excluding radiologists, pathologists and anesthesiologists) within the study period. Individuals were excluded from the study if they were not living in Manitoba for the entire study period, and the year following the study period (to allow for follow-up). Patients whose records only included visits to emergency departments, inpatient hospitalizations, or doctors that were not active throughout the entire three-year study period, as well as those whose only visits were to specialists on referral from another physician, were excluded.

Residents of the three northern-most regional health authorities (RHAs; NOR-MAN, Burntwood and Churchill) were excluded from the study cohort, because our initial analyses indicated significant differences in the patterns of ambulatory care in these RHAs compared to the rest of the province. This is potentially due to the high percent of salaried physicians whose administrative claims are not always captured by the data, working in these areas [21]. Another contributing factor might be the lack of reporting of on-reserve First Nations nursing station visits to nurse practitioners. Our analyses also indicated a significant turnover of physicians practicing in these regions, making the application of the physician assignment algorithm (see below) difficult. In order to avoid introducing bias in our analyses, residents of the “North” (a total of 13,089 people) were excluded from this study as outliers. The final study cohort of Manitobans with chronic diseases included 347,606 individuals.

### Physician assignment algorithm

**Figure 2:**
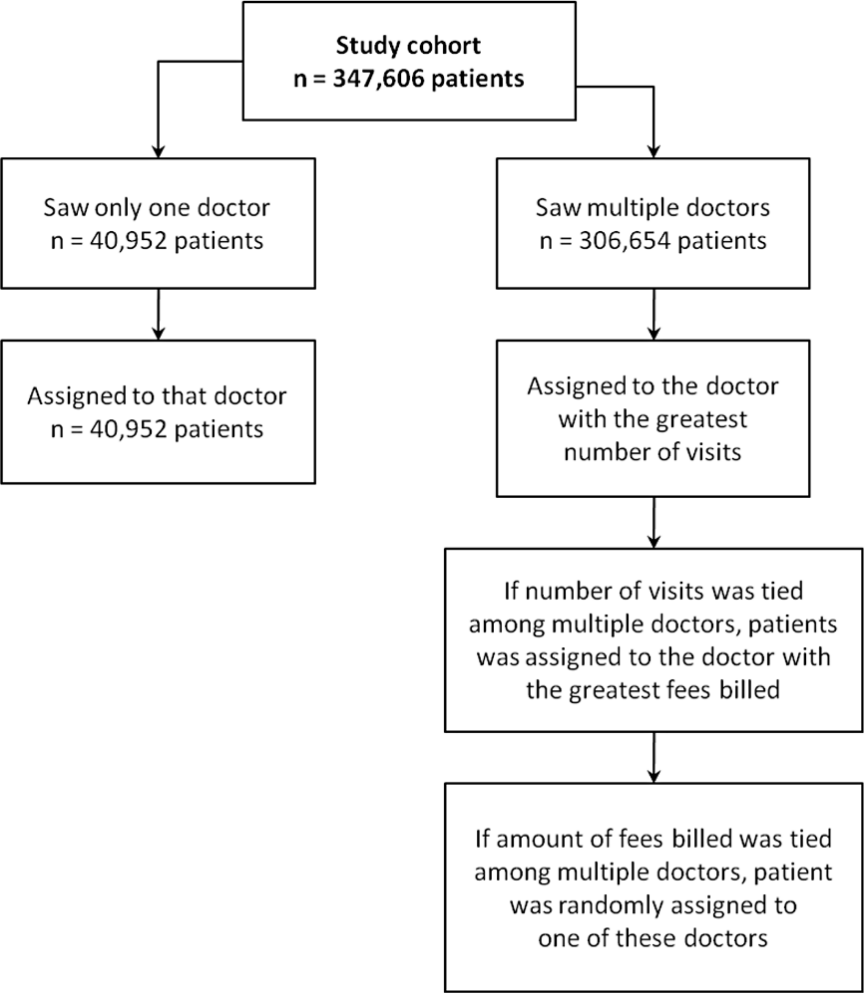
**Physician assignment algorithm.** Patients in the study cohort were assigned to a physician based on the frequency of their physician visits during the 3-year study period.

The physician assignment algorithm (Figure 2) used to assign all individuals in the study cohort to a physician has been applied in numerous previous studies [21–25]. The algorithm is based on the frequency of ambulatory visits the patient has made to each physician. Only patients who had made at least four visits during the three–year study period were assigned to a physician. This study analyzed the choice of doctor patients made when seeking ambulatory care; therefore, prior to physician assignment, all visits that resulted from a referral from one physician to another (as indicated by a referral code in the medical claim) were excluded from the algorithm. We also excluded visits to emergency departments, visits to an inpatient setting, visits for maternity care, and visits to doctors that were not active during the entire study period.

### Ambulatory care visit patterns

Visits to ambulatory care providers were divided into nine categories (Table 1). The first three categories included visits to a primary care physician. The next three categories included visits to a specialist without referral, and the last three categories included specialist visits made on referral. Referrals to specialists were determined from the associated billing codes that increase the value of the visit to the billing physician. We categorized all visits to a specialist within 6 months of a referral billing code as referred visits. The patterns of specialist visits with a referral were divided into categories based on who made the referral. The referring doctor could be a specialist, a primary care physician, or an inpatient/emergency department physician. Although inpatient and emergency department visits were excluded from the main analyses, they were included when determining referrals since these doctors often provide referrals for future ambulatory specialist care.

**Table 1:** **Ambulatory care visit patterns**

***Visits to a primary care physician***	
1 Visits to the assigned PCP*	
2 Visits to a PCP other than the assigned PCP	
3 Visits to a PCP by patients assigned to a specialist	
***Visits to a Specialist without Referral***	
4 Visits to a specialist by patients with an assigned PCP	
5 Visits to the assigned specialist	
6 Visits to another (non-assigned) specialist	
***Visits to a Specialist with Referral***	
7 Visits with referral made by the patient’s assigned PCP	
8 Visits with referral made by another (non-assigned) PCP	
9 Visits with referral made by another specialist	

*PCP: primary care physician.

## Results

### Cohort characteristics

The study cohort included a total of 347,606 patients aged 19 and older. The distribution of the cohort by number of chronic diseases and area of residence (Winnipeg or non–Winnipeg) is presented in Table 2. The majority of study participants in Winnipeg and in the rest of the province had one chronic disease, with an inverse association between number of patients and number of diseases in both groups.

**Table 2:** **Distribution of chronic disease patients by location of residence and number of diseases**

Number of chronic diseases	Winnipeg		Non-Winnipeg		Total number of patients
	#	%	#	%	
	Patients	Patients	Patients	Patients	
**1**	133,405	62.74	79,229	37.26	212,634
**2**	57,634	60.71	37,297	39.29	94,931
**3**	18,720	60.62	12,161	39.38	30,881
**4**	4,535	59.19	3,127	40.81	7,662
**5**	794	57.20	594	42.80	1,388
**6**	65	59.09	45	40.91	110
**Total**	215,153	61.90	132,453	38.10	347,606

### Visit rates and patterns

The total number of visits and visit rates over the three-year study period are presented in Table 3. The average three-year visit rate per patient was similar for Winnipeg and non-Winnipeg residents. For Winnipeggers, 74% of all visits in the study period were to primary care physicians and the remainder were to specialists. In the non-Winnipeg group, 85% of all visits were to primary care physicians. Winnipeg residents visited specialist physicians nearly twice as often as non-Winnipeggers (5.86 visits per patient for Winnipeggers vs. 3.15 visits per patient for non-Winnipeggers over three years).

**Table 3:** **Ambulatory care visit rates of Manitoba chronic disease patients over 3 years by residence area**

	Winnipeg	Non-Winnipeg
**Number of Patients in Residence Area**	215,185	132,421
**Total Ambulatory Care Visits**	4,894,455	2,767,956
**Average Visits per Patient**	22.74	20.90
**Total Visits to Primary Care Physicians**	3,632,747	2,350,554
**Average Visits to Primary Care Physicians per Patient**	16.88	17.75
**Total Visits to Specialists**	1,261,708	417,402
**Average Visits to Specialists per Patient**	5.86	3.15

The visit patterns by patients whose assigned physician was a primary care physician are presented in Table 4. A visit to the assigned primary care physician was the most common route of accessing the ambulatory care system (52.6% for Winnipeggers and 58.3% for non–Winnipeg residents). In both groups, the second most common visit type was accessing a different (non-assigned) primary care physician. Specialist visits represented a greater percent of visits for Winnipeg residents than non-Winnipeg residents, and were equally divided between referred and non–referred visits regardless of geography. Among specialist visits, the most frequent type was to specialists assigned as the principal provider for that patient, representing 8.3% of visits for Winnipeg patients vs. 5.0% for non–Winnipeg residents. Among patients whose assigned physician was a specialist, only 1.4% and 0.4% of visits by Winnipeg and non-Winnipeg residents, respectively, were to a primary care physician.

**Table 4:** Ambulatory care visit and referral patterns of Manitoba chronic disease patients over 3 years by residence area: percent of total visits by patients with an assigned PCP*

Visit category	Winnipeg	Non-Winnipeg
**Visits to Assigned PCP**	**52.55**	**58.29**
**Visits to Another PCP**	**17.31**	**23.10**
**Visits to a Specialist by Referral**	**13.34**	**7.99**
By Assigned PCP	6.15	1.87
By Another PCP	4.81	4.19
By Specialist	2.38	1.93
**Visits to a Specialist without Referral**	**13.40**	**7.82**
Assigned Physician is a PCP	3.51	1.48
Assigned Physician is the Specialist visited	8.33	4.97
Assigned Physician is another Specialist	1.56	1.37

*PCP: primary care physician. Bold text indicates the percent of total visits; regular text is used to describe the referral patterns or assigned physician in each category.

## Discussion

### Main findings of the study

This study examines the ambulatory visit pattern of Manitobans with specific types of chronic disease with the aim of determining whether their care fits within the recommended patterns, including continuity of care with one physician. The majority of visits were to a primary care physician, with just over half of all visits to the assigned primary care physician. However, 26.2% of all visits to a primary care physician in Winnipeg and 28.7% outside of Winnipeg over the three-year study period were not to the assigned primary care physician. These visits seem to represent a lost opportunity for well-coordinated care. Patient access to specialist care without a referral was uncommon (13.7% of visits for Winnipeg residents and 8.0% for non-Winnipeg residents). The highest proportion of specialist visits with referral was with a referral from a non-assigned primary care physician (4.8% of all visits for Winnipeg and 4.2% for non-Winnipeg), particularly outside of Winnipeg where the rate of referral is more than twice as high from non-assigned physicians compared to assigned physicians.

### Is coordination of care supported by the current model of care in Manitoba?

In Canada, all patients are free to choose their own primary care physician. Although direct access to specialists is possible, many provinces encourage access to specialist care via referral from a primary care physician by paying lower fees for non-referred consultations. In this way, primary care physicians act as “gatekeepers” to specialist care (i.e. secondary and tertiary care providers who do not generally have first contact with patients). The rationale for this recommended route of accessing ambulatory care is that it provides optimal continuity of care for the patient. Continuity and coordination of care are widely believed to be essential components of high-quality patient care [26, 27] and have been shown to result in better patient health outcomes [28–30]. We have demonstrated that in Manitoba referrals for specialist consultation frequently originate from primary care physicians who are the not the patient’s regular provider. These providers are less likely to be coordinating the patient’s care, because they have not developed a continuous care relationship with the patient.

Visits of this nature may be generated through a number of circumstances. The non-assigned physician may work in the same clinic as the assigned provider and provide care while the assigned provider is away or not readily available. In this case, the non-assigned provider would likely have access to the patient’s clinical record, ensuring continuity of information [11]. Alternatively the patient may have sought care from a different physician either due to the unavailability of the assigned physician or specifically to obtain the desired specialist referral that the assigned physician may not have felt appropriate or warranted. A non-assigned physician may be more likely to refer a patient for specialist care because of a lack of information about the patient’s health history or previous care-seeking behaviours. Due to data limitations we were unable to determine which scenario is more or less responsible for the high proportion of the referrals that originated with non-assigned physicians.

Thirteen percent and 7.8% of visits to specialists by Winnipeg and non-Winnipeg patients, respectively, were made without any referral at all. Several studies have shown that geography is a factor in patient access to specialist care: urban centres in Canada have ten times as many specialists per capita as rural areas and rural residents are less likely to use specialist services than urban dwellers [31, 32]. Higher socioeconomic status also predicts a higher frequency of specialist visits [33, 34]. When patients visit multiple health care providers or access specialist care directly, communication among providers and between patients and physicians can be a challenge. Several other studies have emphasized the lack of effective communication between primary care physicians and specialists during the often cumbersome process of seeking consultation and integrating a new treatment plan [35, 36]. This difficulty likely exacerbates the loss of coordinated care in situations where patients seek specialist care directly.

### Limitations of the data

The limitations of this study are primarily related to the limitations in administrative claims data for physician visits, since the data used in this study were not developed specifically for research purposes. The study is based on physician claim data, which are submitted following visits to physicians remunerated on a fee-for-service basis. However, some physicians are paid through alternative mechanisms, in which case claims may not be submitted as reliably, and these visits would not have been included in the analysis. The number of visits to primary care physicians outside of Winnipeg is likely to be underestimated, as up to 40% of these physicians are paid via alternative funding arrangements [24]. Previous work has suggested that up to one–third of the visits to alternatively funded physicians may be missing from the claims data [21]. Claims are also missing from primary care physicians in Winnipeg because some (less than 10%) of these are paid via alternative funding mechanisms and because services provided by nurse practitioners are not included during the years of study. We have not adjusted the results to address these gaps in the data but were forced to remove three northern regions with a high rate of alternatively funded primary care physicians, and thus we could not include the entire province’s population. Studies have demonstrated that using the “majority-of-care” criterion for assigning physicians may overestimate the contribution of specialists and thus introduce bias [37]; however, these studies are based out of the U.S. where there is no gatekeeper function of primary care and should be interpreted with caution when examining a Canadian system. This study examines the patterns of physician access and referral in Manitoba, but is not intended to assess the quality of care ambulatory patients receive. It should also be noted that because the organization of the healthcare system differs among provinces and countries, this study has limited generalizability to other regions.

## Conclusion

This population-based study demonstrates that the current primary care system in Manitoba does not fit the recommended patterns of coordination of ambulatory care. The reason for this discrepancy and the impact on the quality of care patients receive are as yet unknown. Other Canadian jurisdictions, including Ontario [3, 38], Quebec [3, 39] and Alberta [40], have moved to formal patient-provider attachment arrangements to support the fundamental principles of high quality primary care. Future research should explore the impact of those initiatives on referral rates and the origin of specialist referrals in those jurisdictions.

## Electronic supplementary material

Additional file 1: **Definition Criteria for Chronic Conditions.** (DOCX 19 KB)

## Abbreviations

MCHP: Manitoba Centre for Health Policy
RHA: Regional health authority.
