# Supplementary material for: Do primary care physicians coordinate ambulatory care for chronic disease patients in Canada?
Source: BMC Fam Pract. 2014 Aug 30;15:148. doi: 10.1186/1471-2296-15-148 (PMC4160545; doi:10.1186/1471-2296-15-148)
Supplement: Supplementary file 1 — Additional file 1: Definition Criteria for Chronic Conditions. (DOCX 19 KB) [file 12875_2014_1121_MOESM1_ESM.docx]

**Additional file 1. Definition Criteria for Chronic Conditions**

| Diabetes Mellitus (Diabetes) |
| --- |
| - At least one hospital diagnosis: diabetes (ICD-10-CA codes: E10-E14) in three years **OR** - At least two ambulatory visit diagnoses: diabetes (ICD-9-CM code: 250) in three years **OR** - At least one prescription: insulin and analogues (ATC code: 10A), blood glucose lowering drugs, excluding prescriptions for insulin (ATC code: 10B) in three years |
| Congestive Heart Failure |
| - At least one hospital diagnosis: heart failure (ICD-10-CA code: I50), congestive heart failure (ICD-10-CA code: I50.0) in three years **OR** - At least three ambulatory visit diagnoses: heart failure (ICD-9-CM codes: 402, 428) in three years |
| Mood Disorders |
| - One hospital diagnosis: mood disorders (ICD-10-CA codes: F36, F33, F38, F38.1), stress and adjustment disorders (ICD-10-CA codes: F43, F43.2, F43.8), mental and behavioural disorders (ICD-10-CA code: F53), emotional disorders (ICD-10 CA code: F93) in three years **OR** - Three ambulatory visit diagnoses: mood disorders (ICD-9-CM code: 296), reaction to stress and adjustment disorders (ICD-9-CM code: 309), depressive disorders (ICD-9-CM code: 311) in three years **OR** - One hospital diagnosis: anxiety disorders (ICD-10-CA codes: F40, F41, F41.1), depressive disorders (ICD-10-CA code: F32), mood disorders (ICD-10-CA code: F34.1), obsessive-compulsive disorders (ICD-10-CA code: F42), dissociative disorders (ICD-10-CA code: F44), somatoform disorders (ICD-10-CA codes: F45.0, F45.1) in three years **AND** at least one prescription: antidepressants and mood stabilizers (ATC codes: N03AB02, N03AB52, N03AF01, N06A) in three years **OR** - Three ambulatory visit diagnoses: anxiety disorders (ICD-9-CM code: 300) in three years **AND** at least one prescription: antidepressants and mood stabilizers (ATC codes: N03AB02, N03AB52, N03AF01, N06A) in three years |
| Ischemic Heart Disease |
| - At least one hospital diagnosis: ischemic heart disease (ICD-10-CA codes: i20-I25) in three years **OR** - At least two ambulatory visit diagnoses: ischemic heart disease (ICD-9-CM codes: 410-414) in three years **OR** - At least one ambulatory visit diagnosis: ischemic heart disease (ICD-9-CM codes: 410-414) in three years **AND** at least two prescriptions: vasodilators (ATC codes: C01DA02, C01DA05, C01DA08, C01DA14), other cardiac drugs (ATC code: C01EB09), beta blocking agents (ATC codes: C07AA02, C07AA03, C07AA05, C07AA06, C07AA12, C07AB02, C07AB03, C07AB04, C07AB07, C07AG01, C07BA05, C07BA06, C07BA12, C07CA03, C07CB03), calcium channel blockers (ATC codes: C08CA01, C08CA02, C08CA04, C08CA05, C08CA06, C08DA01, C08DB01), angiotensin converting enzyme inhibitors (ACEI; ATC codes: C09AA01, C09AA02, C09AA03, C09AA04, C09AA05, C09AA06, C09AA07, C09AA08, C09AA09, C09AA10, C09BA02, C09BA03, C09BA04, C09BA06, C09BA08), angiotensin II antagonists (ATC codes: C09CA01, C09CA02, C09CA03, C09CA04, C09CA06, C09CA07, C09DA01, C09DA02, C09DA03, C09DA04, C09DA06, C09DA07) in three years |
| Total Respiratory Mortality |
| - At least one hospital diagnosis: bronchitis and bronchiolitis (ICD-10-CA codes: J20, J21, J40-J42), emphysema (ICD-10-CA code: J43), chronic airway obstruction (ICD-10-CA code: J44) in three years **OR** - At least one ambulatory visit diagnosis: bronchitis and bronchiolitis (ICD-9-CM codes: 466, 490, 491), emphysema (ICD-9-AM code: 492), asthma (ICD-9-CM code: 493), chronic airway obstruction (ICD-9-CM code: 496) in three years |
| Hypertension |
| - At least one hospital diagnosis: hypertensive diseases (ICD-10-CA codes: I10-I15) in three years **OR** - At least two ambulatory visit diagnoses: hypertensive and chronic kidney diseases (ICD-9-CM codes: 401-405) in three years **OR** - At least two prescriptions: antihypertensives (ATC codes: C02AB01, C02DC01), diuretics (ATC codes: C03BA11, C03DB02), beta blocking agents (ATC codes: C07AA12, C07AB03, C07CA03), calcium channel blocker (ATC code: C08DA01), angiotensin converting enzyme inhibitors (ACEI; ATC codes: C09AA05, C09BA02), angiotensin II antagonists (ATC codes: C09CA01, C09DA01) in three years |
